# Development of a pH-Sensitive Nanoparticle via Self-Assembly of Fucoidan and Protamine for the Oral Delivery of Insulin

**DOI:** 10.3390/pharmaceutics16101323

**Published:** 2024-10-11

**Authors:** Hongying Cai, Fanxing Yong, Rui Li, Jianping Chen, Xiaofei Liu, Bingbing Song, Zhuo Wang, Qiaoli Zhao, Saiyi Zhong

**Affiliations:** 1 Guangdong Provincial Key Laboratory of Aquatic Product Processing and Safety, Guangdong Province Engineering Laboratory for Marine Biological Products, Guangdong Provincial Engineering Technology Research Center of Marine Food, Key Laboratory of Advanced Processing of Aquatic Product of Guangdong Higher Education Institution, Guangdong Provincial Science and Technology Innovation Center for Subtropical Fruit and Vegetable Processing, Guangdong Provincial Engineering Technology Research Center of Prefabricated Seafood Processing and Quality Control, College of Food Science and Technology, Guangdong Ocean University, Zhanjiang 524008, China; 13414866246@163.com (H.C.); 18035535189@163.com (F.Y.); cjp516555989@gdou.edu.cn (J.C.); liuxf169@126.com (X.L.); song@gdou.edu.cn (B.S.); wangzhuo4132@outlook.com (Z.W.); zql2819557995@163.com (Q.Z.); 2 Shenzhen Research Institute, Guangdong Ocean University, Shenzhen 518108, China; 3 Southern Marine Science and Engineering Guangdong Laboratory (Zhanjiang), Zhanjiang 524025, China

**Keywords:** drug delivery, in vitro stability, oral drug, thermodynamic property, diabetic mice

## Abstract

**Objectives**: Oral insulin delivery has received much attention over the past 20 years due to its high compliance. The aim of this study is to prepare nanoparticles for the oral delivery of insulin; **Methods**: Fucoidan and protamine were used to prepare a pH-sensitive nanoparticle via self-assembly. The secondary structure and in vitro stability of the nanoparticles were characterized using FTIR, XRD, ITC, and TEM. the nanoparticles had a controlled release effect on insulin in simulated intestinal fluid. The pre-liminary therapeutic effect on high-fat-fed type 2 diabetic mice; **Results**: When the fucoidan/protamine mass ratio was 10:3 (*w*/*w*), the particle size and zeta potential were 140.83 ± 1.64 nm and −48.13 ± 0.61 mV.The encapsulation efficiency of insulin was 62.97 ± 0.59%. The preliminary therapeutic effect on type 2 diabetic mice showed that the fasting blood glucose of diabetic mice decreased from 10.28 ± 0.88 mmol/L to 9.22 ± 0.64 mmol/L, the area under the curve value of oral glucose tolerance test was reduced by 11.70%, and the insulin se-cretion of diabetic mice was increased by 13.3%; **Conclusions**: The nanoparticles were prepared successfully by self-assembly. The empty and insulin-loaded nanoparticles remained stable in simulated gastric fluid, and the nanoparticles had a controlled release effect on insulin in simulated intestinal fluid. Moreover, insulin-loaded nanoparticles could relieve on type 2 diabetic mice.

## 1. Introduction

Diabetes is a chronic disease and also an independent risk factor for a variety of diseases, which seriously threatens people’s health. Currently, diabetes causes more than 4 million deaths worldwide every year. It is estimated that by 2050, more than 1.31 billion people around the world will be living with diabetes [1]. Long-term obesity often progresses into metabolic syndrome, which leads to type 2 diabetes mellitus (T2DM) [2]. For the treatment of diabetes, the most common mode of insulin administration is injection by far. However, insulin injections may be painful and inconvenient for type 2 patients [3] and, in some cases, could lead to hypoglycemia, local hypertrophy, and fatty deposits at the injection site, which may be life-threatening [4].

Oral administration is a non-invasive and convenient method of administration that can improve patient compliance and is therefore one of the preferred routes of administration for protein drugs [4]. Oral insulin delivery has received much attention over the past 20 years because it mimics the physiological mechanism by which the liver secretes endogenous insulin after gastrointestinal absorption and may protect pancreatic cells from autoimmune destruction [5]. However, due to the poor absorption by intestinal epithelial cells, and rapid degradation by enzymes and gastric acid in the gastrointestinal tract of insulin, oral insulin administration usually has extremely low bioavailability [6]. Ren et al. [7] crosslinked Chlorella vulgaris (CV) and sodium alginate (ALG) to develop an oral insulin delivery platform (CV@INS@ALG) which increased the abundance of probiotic Akkermansia in db/db type 2 diabetic mice, thereby enhancing the insulin sensitivity of mice. Zhou et al. [8] used poly (lactide-co-glycolide) (PLGA) as the backbone and constructed an insulin-loaded nano-delivery system with ionic liquids (IL) and vitamin B12-chitosan (VB12-CS), which decreased the blood glucose level to about 13 mmol/L and increased its relative pharmacological bioavailability up to 31.8% (that of general nanocarriers was 10–20%).

Marine polysaccharides are of great interest in the development of nano-drug carriers due to their abundant sources, good biocompatibility, biodegradability, and bioactivity [9]. Fucoidan, derived from brown algae, has good water solubility and is negatively charged due to the large number of sulfate groups, which promotes the preparation of nanocomplexes between fucoidan and other positively charged molecules [10]. Tsai et al. [3] combined trimethyl chitosan with fucoidan to deliver oral insulin, which enhanced the transepithelial permeation of insulin through the intestinal epithelial cell barrier and inhibited the α-glucosidase activity. Protamine is a highly alkaline protein isolated from the sperm of fish (usually Pacific salmon). Protamine is rich in arginine, which makes up approximately 70% of its residues, and the arginine-rich structure endows protamine with membrane translocation [11] and cell penetration properties [12]. Protamine nanocapsules have been used to enhance the intestinal absorption of insulin and prolong glucose reduction, lasting up to even 24 h in a diabetic rat model [13]. Lu et al. [14] prepared composite nanoparticles using the self-assembly of fucoidan and protamine for the delivery of an anticancer drug adriamycin and found that the enzymatic catalysis in cancer cells and the acidic intracellular microenvironment (pH 4.5–5.5) triggered the release of an anticancer drug with improved inhibitory effects on metastatic breast cancer cells by the nanoparticles. However, the biomedical applications of fucoidan-based nanoparticles encapsulating anti-diabetic drugs are still at the early development stage. Some studies have used fucoidan-based nanoparticles to encapsulate antitumor drugs such as curcumin [15] and doxorubicin [16], while there are no reports on the encapsulation and delivery of insulin by fucoidan/protamine nanoparticles. Therefore, in this study, a pH-sensitive oral delivery vehicle was prepared using fucoidan and protamine; the preparation conditions were optimized; and the average particle size, zeta potential, chemical interactions, secondary structure, thermodynamic property, and microstructure were determined using a nanoparticle size and zeta potential analyzer, Fourier transform infrared transform spectroscopy, circular dichroism, isothermal titration calorimetry, scanning electron microscopy, etc. In addition, the stability and the release properties of insulin encapsulated by fucoidan/protamine nanoparticles in digestive fluids were investigated, and a preliminary investigation of its hypoglycemic effect in an obese mouse model was carried out.

## 2. Materials and Methods

### 2.1. Materials and Reagents

*Undaria pinnatifida* fucoidan A (Mw 23.91 kDa) and B (Mw 10.33 kDa) were purchased from Qingdao Mingyue Seaweed Group (Qingdao, China). *Undaria pinnatifida* fucoidan C (Mw 19.52 kDa), *Fucus vesiculosus* fucoidan D (Mw 11.61 kDa), and protamine (from *salmon*) were purchased from Sigma-Aldrich (St. Louis, USA). *Laminaria japonica* fucoidan E (Mw 7.86 kDa) was purchased from Shanghai Yuanye Biotechnology Co., Ltd. (Shanghai, China). Insulin and the human recombinant were purchased from Yeasen Biotechnology (Shanghai) Co., Ltd. (Shanghai, China).

### 2.2. Preparation of Fucoidan/Protamine Nanoparticles

Fucoidan was dissolved in ultrapure water to prepare the fucoidan solution (4.5 mg/mL). Similarly, different amounts of protamine were dissolved in ultrapure water to prepare the protamine solutions (0.6–4.5 mg/mL). Fucoidan/protamine nanoparticles were prepared by adding the protamine solution into the fucoidan solution based on different fucoidan/protamine mass ratios (0.2–1.0), and the mixture [17] was mixed for 3 min using a Vortex mixer. The preparation conditions were optimized by evaluating the particle size and zeta potential [17].

### 2.3. The Particle Size and Zeta Potential

The particle size and zeta potential of 1-fold diluted samples (original pH) were measured by a Malvern Zetasizer Nano ZS90 (Shanghai Malvern Panaco Co., Ltd., Shanghai, China) with phase analysis light scattering (PALS) and dynamic light scattering (DLS), respectively.

### 2.4. Characterization of Nanocarriers

#### 2.4.1. Fourier Transform Infrared Spectroscopy (FTIR)

The lyophilized samples with protamine, fucoidan, fucoidan/protamine nanoparticles, and the fucoidan solution/protamine solution mixture (fucoidan/protamine physical mixture) were mixed with KBr at a mass ratio of 1:100, respectively. The mixture was analyzed using FTIR (Bruker Tensor Ⅱ, Ettlingen, Germany) from 4000 cm^−1^ to 400 cm^−1^ with a scan number of 32 and a resolution of 4 cm^−1^.

#### 2.4.2. Circular Dichroism (CD)

The CD spectra were measured using a Chirascan V100 spectrometer (Applied Photophysics Ltd., Leatherhead, UK) at a scan rate of 1 nm/s to determine the secondary structure of fucoidan, protamine, and the fucoidan/protamine nanoparticles at a concentration of 0.10 mg/mL The spectra were recorded as ellipticity within 190–260 nm.

#### 2.4.3. X-ray Diffraction (XRD)

An X-ray diffractometer (Rigaku Ultima IV, Tokyo, Japan) was used for the diffraction studies. The lyophilized protamine, fucoidan, and fucoidan/protamine nanoparticles were exposed to a copper Kαl, and the angular range was scanned from 5° to 90° at a rate of 2°/min.

#### 2.4.4. Isothermal Titration Calorimetry (ITC)

The interactions between protamine and fucoidan were analyzed using a Nano ITC isothermal titration calorimeter (TA Instruments, New Castle, DE, USA) at 25 °C. In brief, a 350 μL protamine solution was added to the sample cell to react with fucoidan in a titration needle, and the thermal changes in the reaction were recorded at different time intervals. All solutions were thoroughly degassed before titration.

### 2.5. The In Vitro Stability of Fucoidan/Protamine Nanocarriers

In order to test the stability of fucoidan/protamine nanoparticles, the nanoparticles were incubated for 3 h in simulated gastric fluid (SGF, 1 L SGF consisted of pepsin 3.2 g, NaCl 2.0 g, and adjusted pH to 1.2 with HCl, Shanghai Yuanye Biotechnology Co., Ltd., Shanghai, China), 12 h in simulated intestinal fluid (SIF, 1 L SIF consisted of 10.0 g of, 6.8 g of potassium dihydrogen phosphate, and adjusted pH to 6.8 with NaOH, Shanghai Yuanye Biotechnology Co., Ltd., Shanghai, China), and 12 h in simulated body fluid (SBF, 1 L SBF consisted of 8.035 g NaCl, NaHCO_3_ 0.355 g, KCl 0.225 g, K_2_HPO_4_·3H_2_O 0.231 g, MgCl_2_·6H_2_O 0.311 g, CaCl_2_ 0.292 g, Na_2_SO_4_ 0.072 g, 6.118 g TRIS and pH adjusted to 7.4 with HCl, Shanghai Yuanye Biotechnology Co., Ltd., Shanghai, China). The particle size and zeta potential were determined using the same method as described in Section 3.3, and the characteristic peaks were measured by FTIR (using the method described in Section 3.4.1). The micromorphology was also measured using scanning electron microscopy (SEM) (TESCAN MIRA3, Brno, Czech Republic) at an accelerating voltage of 5 kV. The lyophilized nanoparticle samples (size 0.5 mm × 1.0 mm) were stuck onto a cylindrical aluminum stub using double-sided tape, sputtered with a thin layer of gold in an ion sputter coater (K-450X, EMITECH, Laughton, UK), and placed into an SEM chamber to observe the cross-section of the samples.

### 2.6. Preparation of Insulin-Loaded Fucoidan/Protamine Nanoparticles

The protamine solution was added to the insulin solution with equal volume and mixed for 2 min under magnetic stirring; then, the cocktail was added to the fucoidan solution drop by drop and stirred for 3 min using a magnetic stirrer. The mass ratio of protamine, insulin, and fucoidan in the mixed solution was 1:2:3.

### 2.7. Characterization of Insulin-Loaded Fucoidan/Protamine Nanoparticles

In order to characterize insulin-loaded fucoidan/protamine nanoparticles, the nanoparticles were incubated for 3 h in simulated gastric fluid (SGF, pH 1.2) and 12 h in simulated intestinal fluid (SIF, pH 6.8), respectively. After being filtered through a 0.22 μm membrane, the particle size and zeta potential were measured using the same method as described in Section 3.3, and the secondary structure was determined using FTIR (using the method described in Section 3.4.1). The microstructure of the nanoparticles was visualized using a JEM-1400 plus transmission electron microscope (JEOL Ltd., Tokyo, Japan). The nanoparticles were diluted 2 times with ultrapure water and filtered through a 0.22 μm membrane; then, the filtrate was applied onto a 200-mesh carbon-coated copper grid and dried at room temperature; and then, the sample was stained with a drop of 2% (*w*/*v*) phosphotungstic acid and dried at room temperature. The prepared samples were visualized at 100 kV.

### 2.8. Encapsulation Efficiency (EE) and Loading Capacity (LC)

The EE and LC of insulin in the nanoparticle were determined using a previously described method [18] with minor modifications. In brief, the nanoparticle suspensions loaded with insulin were centrifuged at 15,000 r/min for 30 min, and the supernatant was used to test the amount of unloaded insulin using high-performance liquid chromatography (HPLC, e2695, Waters, Milford, MA, USA). The column was an Agilent ZORBAX Eclipse XDB-C18 (4.6 mm × 250 mm, 5 μm). The mobile phase was a mixture of acetonitrile and 0.1 mol/L sodium dihydrogen phosphate (pH 3.0, 30:70 *v*/*v*) with a flow rate of 1.0 mL/min, and the detection wavelength was 214 nm. The EE and LC were calculated using Equations (1) and (2), respectively.

EE (%) = [1 − (the amount of unloaded insulin/the total amount of added insulin)] × 100%(1)

LC (%) = [(the total amount of added insulin-the amount of unloaded insulin)/the total weight of nanoparticles] × 100% (2)

### 2.9. In Vitro Release Property of Nanoparticle Encapsuled Insulin

Insulin-loaded nanoparticles solutions were added into SGF and SIF at a 1/10 (*w*/*v*) ratio, then kept at 37 °C, and stirred at a speed of 300 r/min, respectively. A solution of 200 μL was taken at different time intervals, centrifuged, and filtered through a 0.22 μm polyethersulfone membrane, and the same amount of SGF or SIF was supplemented to keep a constant volume [19]. The insulin content was determined using HPLC, as described in Section 2.8.

### 2.10. Experimental Animals

#### 2.10.1. Modeling and Grouping

Male C57BL/6J diabetic mice (aged 20–22 weeks) were supplied by the Zhuhai Best Test Co., Ltd. (Zhuhai, China). Before performing the following experiments, all mice were exposed to a pathogen-free environment (temperature: 21 ± 2 °C; relative humidity: 60 ± 10% and 12:12 h light and dark cycles) in acclimatization feeding for one week. After their fasting blood glucose (FBG) was measured, the mice were randomly divided into the model group (fed 60% high-fat diet, D12492, with gavage 10 mL/kg/d saline, n = 6) and the sample group (fed 60% high-fat diet, with gavage 200 mg/kg/d nanoparticles including 41.98 mg/kg/d insulin, n = 6), and the model group was given the same volume of normal saline. The animal experiments were conducted in the Laboratory Animal Center of Guangdong Ocean University and approved by the Laboratory Animal Ethics Review Committee (No. GDOU-LAE-2022-024).

#### 2.10.2. Measurement of Body Weight and Blood Glucose

During the 3 weeks of oral gavage, the animal weight was recorded every 3 d. Before measuring their FBG, the mice were fasted for 12 h and a drop of blood sample was collected from the tail of mice, measured with a commercial blood glucose meter (Shanghai Yuanye Biotechnology Co., Ltd., Shanghai, China). For the oral glucose tolerance test (OGTT), the mice were given 2 g/kg glucose solution by mouth after 12 h of fasting; and the blood glucose values of the mice at 0, 15, 30, 60, 90, and 120 min were recorded; and the area under the curve (AUC) was calculated.

#### 2.10.3. Serum Insulin and Total Cholesterol Assay

The serum insulin level of the mice was determined using an insulin ELISA kit (Jiangsu Meimian Industrial Co., Ltd., Yancheng, China) following the manufacturer’s protocol. The total triglycerides kit (Nanjing Jiancheng Bioengineering Institute, Nanjing, China) was used to detect the serum cholesterol level.

### 2.11. Statistic Analysis

One-way analysis of variance (ANOVA; *p* < 0.05) and Duncan’s multiple range test were carried out to analyze the differences between the samples using Statistic Package for the Social Science software (SPSS 25.0, SPSS Inc., Chicago, IL, USA). The data were expressed as mean ± standard deviation (SD). Three parallel experiments were performed, and all measurements were carried out in triplicate.

## 3. Results

### 3.1. Optimization of the Preparation Conditions of Fucoidan/Protamine Nanoparticles

The fucoidan/protamine nanoparticles were prepared according to Lu et al.’s method [14]. As previous research has not explored the effects of fucoidan from different sources on nanoparticles, we determined the fucoidan: protamine mass ratio and fucoidan source (*Undaria pinnatifida*, *Fucus vesiculosus*, and *Laminaria japonica*) based on particle size and zeta potential. Table 1 shows that when using fucoidan D derived from a *Fucus vesiculosus* source with an Mw of 11.61 kDa and at a protamine: fucoidan mass ratio of 0.3, the prepared nanoparticles had the smallest size of 140.83 ± 1.64 nm and the lowest zeta potential of −48.13 ± 0.61 mV among all the tested groups. Research has shown that when the size is less than 300 nm, nanoparticles can be absorbed by intestinal epithelial cells directly [20]. In addition, the larger the zeta potential absolute value, the stronger the electrostatic repulsion between particles and thus the more stable the system [21]. Therefore, *Fucus vesiculosus* fucoidan (Mw 11.61 kDa) and the protamine: fucoidan mass ratio of 0.3 were chosen for the preparation of the nanoparticles.

### 3.2. Characterization of Fucoidan/Protamine Nanoparticles

#### 3.2.1. Chemical Interactions

**Figure 1:**
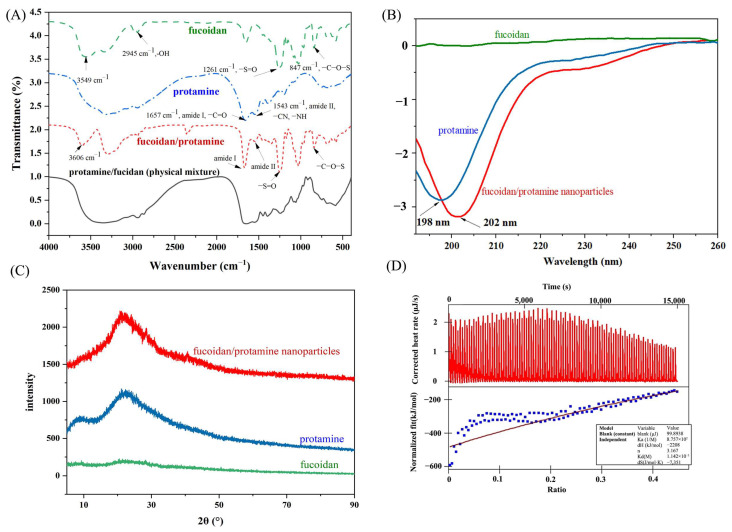
Spectroscopic analysis of fucoidan/protamine nanoparticles. (**A**) Fourier transform infrared spectroscopy (FTIR) spectra of fucoidan, protamine, fucoidan/protamine physical mixture, and fucoidan/protamine nanoparticles; (**B**) circular dichroism (CD) spectra; (**C**) X-ray diffraction (XRD) spectra of fucoidan, protamine, and fucoidan/protamine nanoparticles; (**D**) isothermal titration calorimetry (ITC) data for titration of protamine solution with fucoidan solution.

The chemical interactions between fucoidan and protamine were characterized by FTIR (Figure 1A). Fucoidan showed a typical sulfated polysaccharide signal with characteristic absorption peaks at 3549 cm^−1^ and 2945 cm^−1^ corresponding to the –OH and –CH group, respectively. The absorption bands at 1253 cm^−1^ were caused by C–O–C stretching [22], and the absorption peaks at 1051 cm^−1^ and 821 cm^−1^ indicate the presence of C=O and C-O-S bonds, respectively [23,24]. The absorption peak detected near 1700–1750 cm^−1^ proved the presence of uronic acids in fucoidan molecules [25]. The fucoidan/protamine nanoparticles showed the characteristic peaks of both fucoidan and protamine with red or blue shifts. Specifically, the characteristic peak of the –OH had a red shift to 3606 cm^−1^ of fucoidan/protamine nanoparticles [26,27]. The amide I and amide II absorption peaks of fucoidan/protamine nanoparticles had blue-shifted to 1670 and 1547 cm^−1^ [28,29] from 1657 and 1543 cm^−1^ of protamine, respectively, and the characteristic absorption peaks of fucoidan at 1261 and 847 cm^−1^ had red-shifted to 1250 and 841 cm^−1^, respectively, indicating the formation of a fucoidan/protamine complex through electrostatic interactions [26]. However, the physical mixture showed a superposition of peaks of individual components, similar to that in previous research [30,31].

#### 3.2.2. Secondary Structure

CD was employed to determine the molecular secondary structure. Figure 1B shows the CD spectra of fucoidan, protamine, and fucoidan/protamine nanoparticles. There was no obvious peak of fucoidan from 190 nm to 260 nm, which was consistent with our previous studies [32,33]. Protamine showed a random coil structure demonstrated by a negative peak at 198 nm [14]. The formation of fucoidan/protamine complex nanoparticles was indicated by the red shift in the negative band of protamine at 198 nm to 202 nm and increased ellipticity. Previous research has shown that protamine can form α-helix conformation with a transfer RNA [34], indicating that protamine contributed to α-helical conformation. However, the α-helix structure was not typical in the fucoidan/protamine nanoparticles of this study.

#### 3.2.3. Diffraction Property

XRD was used to characterize the amorphous and crystalline formation of the macromolecules, and the XRD patterns of fucoidan, protamine, and fucoidan/protamine nanoparticles are shown in Figure 1C. The XRD curve of fucoidan showed a weak diffraction peak at 2θ = 21.2°, in agreement with a previous study, which has reported that most polysaccharides exhibit diffraction peaks at about 2θ = 20° [35], while protamine had two broad peaks at 2θ = 9.2° and 2θ = 22.3°, respectively, indicating that protamine is a semicrystalline substance. The XRD curve of nanoparticles had only one broad-like peak at 2θ = 22.1°, indicating that fucoidan/protamine nanoparticles were amorphous [36].

#### 3.2.4. Thermodynamic Property

ITC has been widely used to research the thermodynamics of the reaction between two oppositely charged polyelectrolytes. The ITC original data (top) and area integration (bottom) are shown in Figure 1D. During the process, the protamine solution with a concentration of 1.5 mg/mL was added dropwise to 4.5 mg/mL fucoidan solution. We can see that the whole reaction was divided into two steps in Figure 1D: the first step was an exothermic reaction (before around 7000 s), the second step was an endothermic reaction (after around 7000 s), and the first step was the reaction of the binding of fucoidan and protamine, so we only performed data fitting for the first step. The binding affinity was generally judged by the equilibrium dissociation constant (K_d_), which has been used to assess the strength of the bimolecular interaction [37]. The smaller the K_d_ value, the greater the binding affinity between the substances, and vice versa. The K_d_ of the sample obtained in the experiment was 1.14 × 10^−3^, indicating that fucoidan and protamine had a good binding ability. Research has shown that when the binding energy is below −29.3 kJ/mol, the two polymers have a strong binding force [38]. An exothermic enthalpy of −2.21 × 10^3^ kJ/mol indicated a strong interaction between fucoidan and protamine. Furthermore, there was a non-specific binding mode between the protamine and fucoidan molecules demonstrated by the presence of multiple binding sites (n = 3.17), which was about half the number of a previous study [14]; this might be due to the different sources, molecular weights and sulfate contents of fucoidan.

### 3.3. In Vitro Stability of Fucoidan/Protamine Nanoparticles

#### 3.3.1. Particle Size and Zeta Potential of Fucoidan/Protamine Nanoparticles

**Figure 2:**
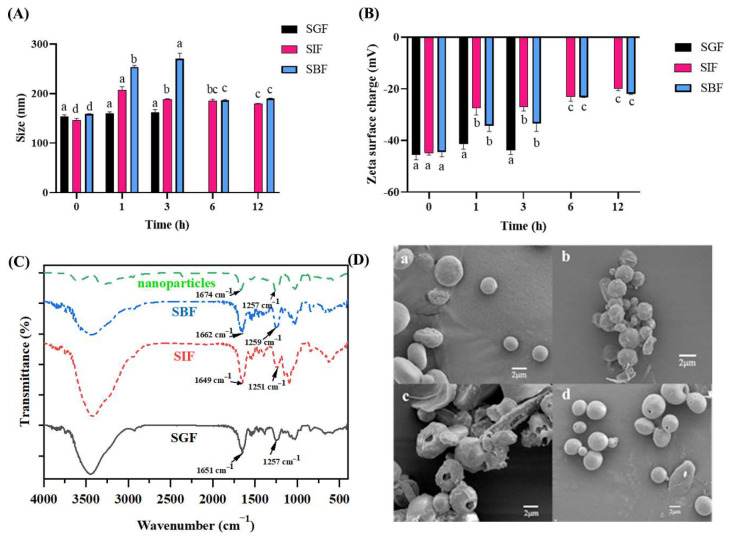
The in vitro stability of fucoidan/protamine nanoparticles in different digestive fluids. (**A**) The particle size and (**B**) zeta potential of fucoidan/protamine nanoparticles after incubation in simulated gastric fluid (SGF, pH 1.2), simulated intestinal fluid (SIF, pH 6.8), and simulated body fluid (SBF, pH 7.4), respectively. (**C**) The FTIR spectra of original fucoidan/protamine nanoparticles and the nanoparticles incubated in SGF, SIF, and SBF for 3 h, 12 h, and 12 h, respectively. (**D**) The micro morphologies of fucoidan/protamine nanoparticles. The scanning electron microscope (SEM) images of (**a**) original fucoidan/protamine nanoparticles and the nanoparticles incubated in (**b**) SGF for 3 h, (**c**) SIF for 12 h, and (**d**) SBF for 12 h. The different lowercase letters above the columns indicate statistical differences in the same digestive liquid at *p*  <  0.05.

The nanoparticles were prepared under the same conditions, and there was no significant difference in the particle size, potential, and other parameters of the nanoparticles in different buffers initially. The in vitro stability of the fucoidan/protamine nanoparticles was determined after exposure to digestive fluids. Figure 2A showed that after incubation in SGF (pH 1.2) for 3 h, the particle size of the nanoparticles had no significant change, and the size was lower than 300 nm [20]. The zeta potentials all showed negative values, which might be due to the fucoidan used in this study having a high sulfate content (47.44 ± 3.24%, *w*/*w*) [39], which also contributed to the electrostatic interactions between fucoidan and protamine molecules. Specifically, the zeta potential increased from −45.43 ± 1.64 mV of the original nanoparticles to −43.7 ± 1.45 mV incubated for 3 h in SGF (*p* < 0.05) (Figure 2B). This result might be due to the fact that the low pH value of SGF caused a decline in the surface charge of the complexes. Moreover, various ionic compositions presented in the SGF might result in electrostatic screening effects and the adsorption of counterions, leading to a decrease in the electrostatic repulsion between particles. Meanwhile, the structure of the complexes was gradually destroyed by enzymatic hydrolysis [40].

When incubated in SIF (pH 6.8), the particle size of the nanoparticles increased significantly from 146.4 ± 2.88 nm to 207.3 ± 5.35 nm in the first 3 h (*p* < 0.05) and then decreased significantly in the next 6 h (Figure 2A). The zeta potential significantly increased from −44.77 ± 0.69 mV to −27.5 ± 2.08 mV at 3 h, then decreased to −23.07 ± 1.38 mV at 6 h (*p* < 0.05), and was kept stable until 12 h (Figure 2B). These phenomena might be attributed to the fact that the nanoparticles were gradually dissociated by pancreatin and formed new complexes or micelles composed of bile salts, proteins, and peptides [41].

In SBF, the particle size increased from 158.87 ± 0.45 nm at the beginning to 253.5 ± 2.77 nm at 3 h and decreased at 6 h (Figure 2A). The changes in zeta potential were similar to those in SIF, except that after incubation for 6 h, the zeta potential decreased significantly to −21.9 ± 0.22 mV at 12 h from −44.43 ± 1.48 mV at the original zeta potential (*p* < 0.05) (Figure 2B). The results indicate that the fucoidan/protamine nanoparticles were pH-responsive and they were more stable in the simulated gastric acid environment but easily disrupted in the simulated intestinal environment, thus having the possibility of being used for intestinal targeted drug delivery systems.

#### 3.3.2. FTIR of Fucoidan/Protamine Nanoparticles

To detect the structural change in the nanoparticles after incubation in different digestive fluids, the FTIR spectra (Figure 2C) were obtained using 2 mg lyophilized samples after incubation of the fucoidan/protamine nanoparticles in SGF for 3 h, and in SIF and SBF for 12 h, respectively. When the nanoparticles were incubated in SGF for 3 h, the amide I band had a peak at 1257 cm^−1^, while it shifted to 1251 cm^−1^ and 1259 cm^−1^ when incubated in SIF and SBF for 12 h, respectively. It has been shown that at SGF (pH 6.8), the sulfate ester shoulder of the S=O stretching at 1250 cm^−1^ shifts to 1238 cm^−1^ [42].

#### 3.3.3. Microscopic Morphologies

SEM was used to observe the microscopic morphology change in the samples’ exposure to different digestive fluids, and the results are shown in Figure 2D. The original fucoidan/protamine nanoparticles showed a smooth spherical shape, which endowed them with good dispersibility [43]. After 3 h of incubation in SGF at pH 1.2 (Figure 2D(b)), wrinkles appeared on the surface of the nanoparticles, but there were no pores on the nanoparticle surface. However, after 12 h of incubation in the SIF (pH 6.8) (Figure 2D(c)), many holes appeared on the surface of the nanoparticles, indicating that the complex structure between protamine and fucoidan was greatly disrupted. After incubation in the SBF for 12 h (Figure 2D(d)), the nanoparticles still maintained their original appearance, and the surface of the nanoparticles was smooth without obvious wrinkles and damage. The result was consistent with the infrared spectra, indicating that the nanoparticles were more stable in the simulated gastric acid environment and the body fluid, while easily disrupted in the simulated intestinal environment.

### 3.4. Characterization and In Vitro Stability of Insulin-Loaded Fucoidan/Protamine Nanoparticles

#### 3.4.1. Particle Size and Zeta Potential of Insulin-Loaded Fucoidan/Protamine Nanoparticles

**Figure 3:**
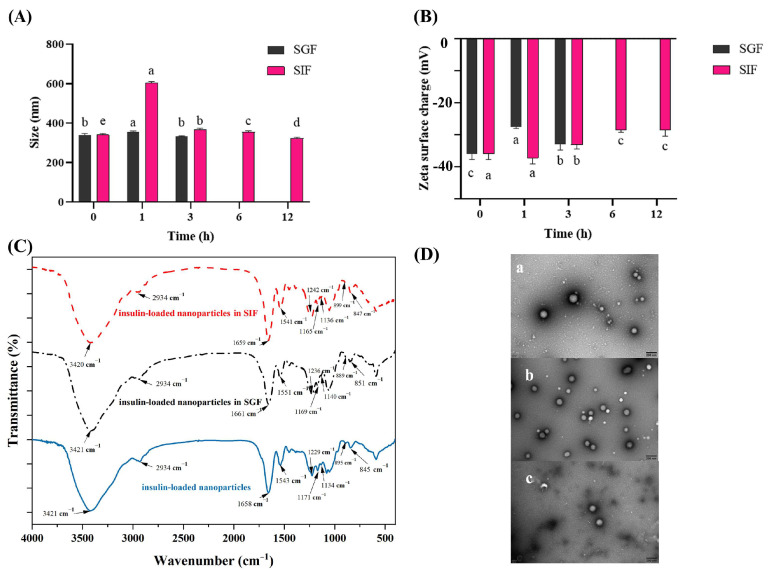
In vitro stability of insulin-loaded fucoidan/protamine nanoparticles in different simulated digestive fluids. (**A**) The particle size and (**B**) zeta potential of insulin-loaded fucoidan/protamine nanoparticles after incubation in simulated gastric fluid (SGF, pH 1.2) for 3 h and simulated intestinal fluid (SIF, pH 6.8) for 12 h, respectively. (**C**) The FTIR spectra of insulin-loaded fucoidan/protamine nanoparticles incubated in SGF and SIF for 3 h and 12 h, respectively. (**D**) The micro morphologies of the insulin-loaded fucoidan/protamine nanoparticles. The transmission electron microscope (TEM) images of (**a**) original insulin-loaded fucoidan/protamine nanoparticles and the nanoparticles incubated in (**b**) SGF for 3 h and (**c**) SIF for 12 h. The different lowercase letters above the columns indicate statistical differences at *p* <  0.05 in the same digestive liquid.

We prepared the fucoidan/protamine nanoparticles, which were expected to be stable in gastric fluid and can rupture in simulated intestinal fluid. In gastric fluid, the particle size had no significant differences within 3 h, while the particle size had a significant difference in intestinal fluid within 1 h, which showed that the nanoparticles were stable in gastric fluid and sensitive in intestinal fluid. For zeta potential, it had a significant difference within 6 h in intestinal fluid, and the digestion time in intestinal fluid reached 6 h or longer. The SEM images in Figure 2D also certify that the nanoparticles were stable in gastric fluid and unstable in intestinal fluid. Meanwhile, there was no significant difference in the initial values of the nanoparticles under different simulated digest conditions, so we only analyzed the significant difference in nanoparticles in the same simulated digest. The particle size of the nanoparticles without insulin was about 140–160 nm; the size of the nanoparticles after insulin loading increased significantly, with a particle size of about 340 nm; and the zeta potential also increased from −44 mV to −35 mV. To test whether the loading of insulin affects the in vitro stability of nanoparticles, the insulin-loaded nanoparticles were incubated in the digestive fluids for different time periods. After exposure to SGF for 3 h, the particle size of the insulin-loaded nanoparticles had no significant change (*p* > 0.05), and the particle size was 332.70 ± 2.54 nm at 3 h (Figure 3A). However, when incubated in SIF for 1 h, this was where we expected it to be released or be disrupted; the particle size increased significantly from 340.30 ± 5.83 nm to 605.30 ± 3.51 nm (*p* < 0.05) and then decreased to 366.53 ± 5.60 nm at 3 h, which might be due to the rupture of some nanoparticles under this condition. The particle size was 322.47 ± 4.79 nm at 12 h (Figure 3A). The zeta potential of the insulin-loaded nanoparticles changed from −36.03 ± 1.39 mV to −32.97 ± 1.47 mV incubated in SGF for 3 h. The zeta potential increased significantly after 3 h of incubation from −36.03 ± 1.39 mV to −33.17 ± 1.03 mV and kept increasing to −28.6 ± 1.51 mV after exposure to SIF for 12 h (*p* < 0.05) (Figure 3B). The results showed that the insulin-loaded nanoparticles still had pH sensitivity and might be unstable in SIF.

#### 3.4.2. FTIR of Insulin-Loaded Fucoidan/Protamine Nanoparticles

The FTIR spectra of the insulin-loaded nanoparticles possessed all the characteristic peaks compared with that of the initial nanoparticles (Figure 3C). When insulin was loaded on the nanoparticles, it was found that the intensity of the absorption peak of S=O at 1229 cm^−1^ was significantly lower than that of the unloaded insulin. It was found that the peak intensity of the nanoparticles decreased at around 1136 cm^−1^ and 899 cm^−1^, which was related to the destruction of fucoidan glycosidic bonds exposed to the SIF [44,45].

#### 3.4.3. TEM Images

TEM was used to analyze the microscopic morphology of nanoparticles loaded with insulin (Figure 3D). The nanoparticles were initially spherical due to surface tension [46]. Importantly, the TEM images showed that the nanoparticles maintained an intact structure after incubation in SGF for 3 h, whereas the surface of the nanoparticles exposed to SIF for 12 h was broken. The TEM images indicated that the stability of the insulin-loaded nanoparticles in gastrointestinal fluids was the same as that of empty nanoparticles.

### 3.5. The In Vitro Release Properties of Insulin

**Figure 4:**
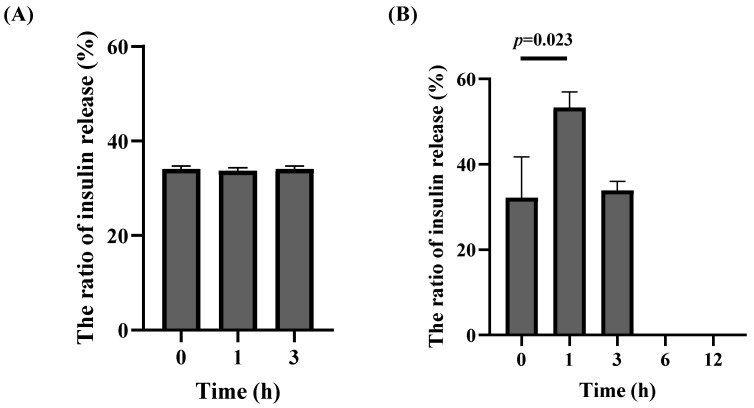
In vitro release ratio of insulin encapsuled in nanoparticles in (**A**) simulated gastric fluid (SGF, pH 1.2) in 3 h and (**B**) simulated intestinal fluid (SIF, pH 6.8) in 12 h.

The EE and LC of insulin were measured using HPLC, which were 65.06% ± 0.59%, and 13.01% ± 0.12%, respectively. The initial insulin concentration loaded in nanoparticles was 650.62 μg/mL, and when exposed to SGF for 3 h, there was no significant change in the release content of insulin encapsulated in the nanoparticles in the first 1 h. At 3 h, the release amount of insulin encapsuled in the nanoparticles was only 34.08% ± 0.57% (Figure 4A), and the retention rate of insulin in the nanoparticles was up to 65.96%. When incubated with SIF, the release amount of insulin encapsulated in the nanoparticles was 32.15% ± 7.83% at the beginning, and the release ratio was 53.50% ± 3.00% and 33.88% ± 1.75% at 1 h and 3 h, respectively, but we could not detect the concentration of the released insulin after 6 h, which might be due to the fact that it was digested by the SIF. Therefore, the results of the in vitro simulated digestion showed that insulin encapsulated in nanoparticles could reduce its loss when exposed to SGF and had a certain controlled release effect on insulin in SIF. In the early detection process, we detected the release behavior of insulin in insulin-loaded nanoparticles every 0.5 h in simulated gastric juice, and there was no significant difference between 0 and 3 h.

### 3.6. Type 2 Diabetic Mice

**Figure 5:**
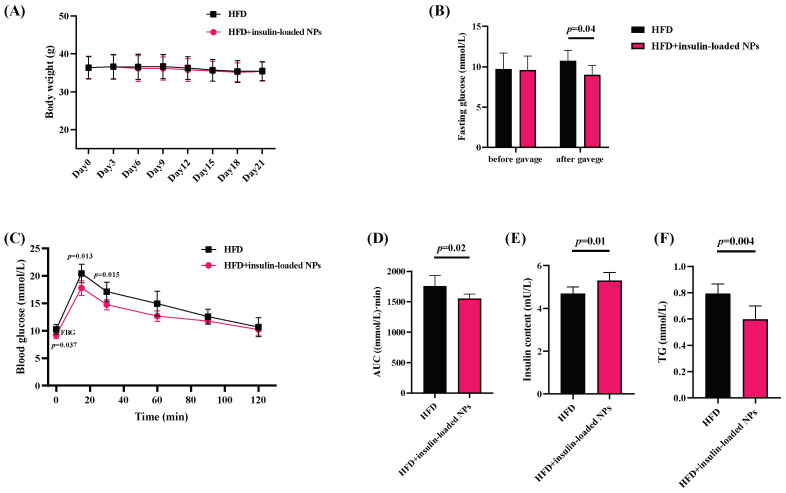
The various indexes of the type 2 diabetic mice treated with high-fat feeding (HFD) and HFD + insulin-loaded fucoidan/protamine nanoparticles (insulin-loaded NPs) for 21 d, respectively, n = 6. (**A**) Changes in body weight. (**B**) Fasting blood glucose before/after savage insulin-loaded NPs. (**C**) Oral glucose tolerance test (OGTT). (**D**) Area under the curve (AUC) value. (**E**) Serum insulin content. (**F**) Total triglyceride content.

Type 2 diabetes was induced in C57BL/6J mice via a high-fat diet (HFD) for 3 months. Figure 5A shows the changes in body weight after 21 d of oral administration of insulin-encapsuled fucoidan/protamine nanoparticles; there was no significant difference between mice fed with HFD and HFD + nanoparticles. Figure 5B shows the changes in fasting blood glucose (FBG) with time, and the insulin-loaded nanoparticles significantly reduced the FBG from 10.28 ± 0.88 mmol/L to 9.22 ± 0.64 mmol/L in diabetic mice (*p* < 0.05). OGTT is a standard method to assess an individual’s ability to regulate blood glucose and is used to understand pancreatic *β*-cell function [47]. The changes in insulin encapsuled nanoparticles on OGTT are shown in Figure 5C, which show that the blood glucose level of mice in the HFD group was significantly higher than that of the HFD + insulin-loaded nanoparticle-treated mousee group at the first 40 min. After overnight fasting, the diabetic mice were fed a glucose solution. The area under the glucose curve (AUC) is an indicator of the total glucose drift after glucose intake and has been widely used to calculate the glycemic index [48]. The results show that the insulin-loaded nanoparticles had a significant control effect on OGTT in the first 40 min of glucose intake in the diabetic mice and significantly reduced the AUC value of OGTT (*p* < 0.05). Type 2 diabetic mice are unable to secrete adequate amounts of insulin, and Figure 5E shows that the serum insulin levels were increased by administering insulin-loaded fucoidan/protamine nanoparticles to the mice (from 4.68 ± 0.32 mU/L to 5.30 ± 0.38 mU/L), increased by 13.30%. Studies have shown that more than 30% of diabetic patients have atherogenic dyslipidemia [49], so to control the total triglyceride content is also very important, and the results indicate that the insulin-loaded nanoparticles could reduce the total triglyceride content of diabetic mice up to 24.49% (Figure 5F) (*p* < 0.05).

## 4. Discussion

Currently, oral administration is a method of administration with good patient compliance, but the oral availability of protein substances is extremely low because proteins are structurally unstable in the gastrointestinal tract and have difficulty penetrating cell membranes. Fucoidan has been extensively studied as a delivery vehicle to improve the bioavailability of oral drugs, and protamine, as a powerful cell-penetrating peptide, plays a significant role in oral drug delivery. In this study, fucoidan derived from *Fucus vesiculosus* and protamine from Atlantic salmon were used to prepare nanoparticles by electrostatic interactions, and when the mass ratio of protamine to fucoidan was 0.3 (*w*/*w*), the nanoparticles had the smallest in size (140.83 ± 1.64 nm) and a high absolute value of zeta potential (−48.13 ± 0.61 mV). Intestinal absorption is an important step in oral insulin delivery, and in general, nanoparticles with a particle size of less than 200 nm and a negative charge on the surface can achieve circulation in the body for longer periods of time [50,51]. Therefore, the surface properties suggest that fucoidan/protamine NPs can be well absorbed by the intestine. Interestingly, in our inquiry, it was found that the fucoidan/protamine nanoparticles were pH-sensitive, and their structure was instable when exposed to SIF (pH 6.8), while they maintained an intact surface in SGF (pH 1.2) and SBF (pH 7.4). Insulin-loaded fucoidan/protamine nanoparticles had similar stability in SGF and SIF, which played a certain protective role for insulin in SIF. The oral delivery of insulin-loaded nanoparticles could reduce FBG and OGTT, increase serum insulin levels, and reduce the total triglyceride content in diabetic mice.

It is reported that the structure of raw materials is often modified to improve the effect of insulin loading and controlled release [52,53]. However, the safety of its chemical modification is not clear in applications, and the safety of substances obtained from raw materials through self-assembly is significantly higher in comparison. At the same time, it is a recognized effective treatment compared to subcutaneous insulin, but it lasts for a shorter period of time [54]. fucoidan/protamine NPs also have a certain improvement effect on cholesterol in high-fat-fed mice. Therefore, this novel nanocarrier was pH-responsive, has great potential in the treatment of diabetes, and might be used as a novel drug carrier for oral administration. Although the insulin-loaded nanoparticles group was able to improve the maximum blood glucose value of OGTT in diabetic mice, unfortunately, whether insulin-loaded NPs can improve insulin resistance and the mechanism of nanoparticles improving the insulin secretion ability of diabetic mice still need to be further explored. In addition, in the preparation of nanoparticles, the ionic interactions between fucoidan and protamine in the preparation of nanoparticles can be analyzed by molecular docking to analyze the characteristic groups of electrostatic interactions and elucidate their action sites for further study.

## 5. Conclusions

In summary, fucoidan and protamine nanoparticles were successfully prepared by self-assembly and loaded with insulin. The results showed that insulin-loaded nanoparticles could be stable in gastric juice and effectively released in intestinal fluid when simulated digestion in vitro, as our expectations, and it could alleviate type 2 diabetic mice by reducing fasting blood glucose values. However, further research is needed on the site of fucoidan and protamine during binding, and the mechanism of insulin-loaded nanoparticles to improve insulin secretion in type 2 diabetic mice.

## Figures and Tables

**Table 1:** Particle size and zeta potential of fucoidan/protamine nanoparticles prepared using different sources of fucoidan and different protamine: fucoidan mass ratios.

Fucoidan Source	Fucoidan-to-Protamine Mass Ratio	Particle Size (nm)	Zeta Potential (mV)
A	10:1	202.83 ± 0.77 ^E^	−24.97 ± 0.69 ^B^
B		234.07 ± 6.55 ^D^	−18.00 ± 0.14 ^A^
C		275.47 ± 2.58 ^B^	−46.33 ± 0.26 ^C^
D		260.10 ± 4.06 ^C^	−48.90 ± 0.73 ^D^
E		320.50 ± 1.08 ^A^	−45.00 ± 0.50 ^C^
A	10:3	251.23 ± 5.33 ^C^	−19.57 ± 0.42 ^B^
B		227.63 ± 2.62 ^D^	−15.53 ± 0.38 ^A^
C		437.03 ± 2.39 ^A^	−44.10 ± 0.93 ^E^
D		140.83 ± 1.64 ^E^	−48.13 ± 0.61 ^D^
E		398.50 ± 7.32 ^B^	−41.70 ± 0.86 ^C^
A	10:5	280.00 ± 2.21 ^C^	−18.90 ± 0.16 ^B^
B		244.97 ± 4.94 ^D^	−15.13 ± 0.21 ^A^
C		404.57 ± 2.12 ^A^	−41.40 ± 0.43 ^D^
D		220.47 ± 1.96 ^E^	−42.00 ± 1.04 ^D^
E		308.87 ± 4.43 ^B^	−37.27 ± 0.91 ^C^
A	10:7	231.50 ± 3.96 ^E^	−20.73 ± 0.87 ^B^
B		293.77 ± 22.33 ^C^	−12.67 ± 0.29 ^A^
C		598.83 ± 18.27 ^A^	−39.67 ± 1.03 ^E^
D		257.43 ± 2.50 ^D^	−30.53 ± 0.87 ^D^
E		447.37 ± 9.52 ^B^	−26.27 ± 0.85 ^C^
A	10:9	241.23 ± 12.97 ^C^	−24.97 ± 0.34 ^B^
B		218.53 ± 2.42 ^D^	−12.30 ± 0.22 ^A^
C		327.57 ± 3.56 ^B^	−34.00 ± 1.18 ^D^
D		194.77 ± 2.16 ^E^	−27.40 ± 0.14 ^C^
E		468.73 ± 2.2 ^A^	−26.63 ± 0.68 ^C^

Note: fucoidan A, *Undaria pinnatifida* (Mw 23.91 kDa); fucoidan B, *Undaria pinnatifida* (Mw 10.33 kDa); fucoidan C, *Undaria pinnatifida* fucoidan C (Mw 19.52 kDa); fucoidan D, *Fucus vesiculosus* (Mw 11.61 kDa); fucoidan E, *Laminaria japonica* (Mw 7.86 kDa). Different letters indicate significant differences at *p* < 0.05 (the uppercase letters compare the groups of fucoidan from different sources with the same fucoidan: protamine mass ratio).

## Data Availability

The original contributions presented in the study are included in this article; further inquiries can be directed to the corresponding authors.

## References

[1] Ong K.L., Stafford L.K., McLaughlin S.A., Boyko E.J., Vollset S.E., Smith A.E., Dalton B.E., Duprey J., Cruz J.A., Hagins H. (2023). Global, regional, and national burden of diabetes from 1990 to 2021, with projections of prevalence to 2050: A systematic analysis for the Global Burden of Disease Study 2021. Lancet.

[2] Heydemann A. (2016). An Overview of Murine High Fat Diet as a Model for Type 2 Diabetes Mellitus. J. Diabetes Res..

[3] Tsai L.C., Chen C.H., Lin C.W., Ho Y.C., Mi F.L. (2019). Development of mutlifunctional nanoparticles self-assembled from trimethyl chitosan and fucoidan for enhanced oral delivery of insulin. Int. J. Biol. Macromol..

[4] Li X., Fu M., Wu J., Zhang C.Y., Deng X., Dhinakar A., Huang W.L., Qian H., Ge L. (2017). pH-sensitive peptide hydrogel for glucose-responsive insulin delivery. Acta Biomater..

[5] Moonschi F.H., Hughes C.B., Mussman G.M., Fowlkes J.L., Richards C.I., Popescu I. (2018). Advances in micro- and nanotechnologies for the GLP-1-based therapy and imaging of pancreatic beta-cells. Acta Diabetol..

[6] Chen M.C., Mi F.L., Liao Z.X., Hsiao C.W., Sonaje K., Chung M.F., Hsu L.W., Sung H.W. (2013). Recent advances in chitosan-based nanoparticles for oral delivery of macromolecules. Adv. Drug Deliv. Rev..

[7] Ren C.J., Zhong D.N., Qi Y.C., Liu C.Y., Liu X.Y., Chen S.H., Yan S., Zhou M. (2023). Bioinspired pH-Responsive Microalgal Hydrogels for Oral Insulin Delivery with Both Hypoglycemic and Insulin Sensitizing Effects. ACS Nano.

[8] Zhou J., Zhang J., Sun Y.W., Luo F.S., Guan M., Ma H.L., Dong X.M., Feng J.F. (2023). A nano-delivery system based on preventing degradation and promoting absorption to improve the oral bioavailability of insulin. Int. J. Biol. Macromol..

[9] Tran P.H.L., Duan W., Tran T.T.D. (2020). Fucoidan-based nanostructures: A focus on its combination with chitosan and the surface functionalization of metallic nanoparticles for drug delivery. Int. J. Pharm..

[10] Tai M.R., Ji H.W., Chen J.P., Liu X.F., Song B.B., Zhong S.Y., Rifai A., Nisbet D.R., Barrow C.J., Williams R.J. (2023). Biomimetic triumvirate nanogel complexes via peptide-polysaccharide-polyphenol self-assembly. Int. J. Biol. Macromol..

[11] Kauffman W.B., Fuselier T., He J., Wimley W.C. (2015). Mechanism matters: A taxonomy of cell penetrating peptides. Trends Biochem. Sci..

[12] Wu J.M., Jones N., Fayez N.A.L., Chao P.H., Wu A., de Araujo D.R., Rouhollahi E., Jia A., Li S.D. (2023). Protamine-mediated efficient transcellular and transmucosal delivery of proteins. J. Control. Release.

[13] Thwala L.N., Delgado D.P., Leone K., Marigo I., Benetti F., Chenlo M., Alvarez C.V., Tovar S., Dieguez C., Csaba N.S. (2018). Protamine nanocapsules as carriers for oral peptide delivery. J. Control. Release.

[14] Lu K.Y., Li R., Hsu C.H., Lin C.W., Chou S.C., Tsai M.L., Mi F.L. (2017). Development of a new type of multifunctional fucoidan-based nanoparticles for anticancer drug delivery. Carbohydr. Polym..

[15] Huang Y.C., Kuo T.H. (2016). O-carboxymethyl chitosan/fucoidan nanoparticles increase cellular curcumin uptake. Food Hydrocoll..

[16] Lee K.W., Jeong D., Na K. (2013). Doxorubicin loading fucoidan acetate nanoparticles for immune and chemotherapy in cancer treatment. Carbohydr. Polym..

[17] An J.J., Liu M.Z., Din Z.U., Xie F., Cai J. (2023). Toward function starch nanogels by self-assembly of polysaccharide and protein: From synthesis to potential for polyphenol delivery. Int. J. Biol. Macromol..

[18] Chen H.B., Zhu H.D., Zheng J.N., Mou D.S., Wan J.L., Zhang J.Y., Shi T.L., Zhao Y.J., Xu H.B., Yang X.L. (2009). Iontophoresis-driven penetration of nanovesicles through microneedle-induced skin microchannels for enhancing transdermal delivery of insulin. J. Control. Release.

[19] Lin H.R., Zhang J., Yu C.C., Lu Y., Ning J., Le S.X., Li Y., Zang L.Q. (2019). Oral delivery of insulin via mesoporous carbon nanoparticles for colonic release allows glycemic control in diabetic rats. Carbon Lett..

[20] Epple M., Rotello V.M., Dawson K. (2023). The Why and How of Ultrasmall Nanoparticles. Acc. Chem. Res..

[21] Ghadimi A., Saidur R., Metselaar H.S.C. (2011). A review of nanofluid stability properties and characterization in stationary conditions. Int. J. Heat Mass Transf..

[22] Yuen S.N., Choi S.M., Phillips D.L., Ma C.Y. (2009). Raman and FTIR spectroscopic study of carboxymethylated non-starch polysaccharides. Food Chem..

[23] Lee M.C., Huang Y.C. (2019). Soluble eggshell membrane protein-loaded chitosan/fucoidan nanoparticles for treatment of defective intestinal epithelial cells. Int. J. Biol. Macromol..

[24] Sun X.X., Liu C., Omer A.M., Yang L.Y., Ouyang X.K. (2019). Dual-layered pH-sensitive alginate/chitosan/kappa-carrageenan microbeads for colon-targeted release of 5-fluorouracil. Int. J. Biol. Macromol..

[25] Zeng W.C., Zhang Z., Gao H., Jia L.R., Chen W.Y. (2012). Characterization of antioxidant polysaccharides from *Auricularia auricular* using microwave-assisted extraction. Carbohydr. Polym..

[26] Liu Q.Y., Qin Y., Chen J.J., Jiang B., Zhang T. (2021). Fabrication, characterization, physicochemical stability and simulated gastrointestinal digestion of pterostilbene loaded zein-sodium caseinate-fucoidan nanoparticles using pH-driven method. Food Hydrocoll..

[27] Coimbra M.A., Barros A., Rutledge D.N., Delgadillo I. (1999). FTIR spectroscopy as a tool for the analysis of olive pulp cell-wall polysaccharide extracts. Carbohydr. Res..

[28] Ma T.M., Huang R.H., Collins V.I., Wikstro M., Batabyal D. (2023). Advancement of microfluidic modulation spectroscopy as a highly sensitive protein characterization technique for the detection of small structural changes. Anal. Biochem..

[29] Andonegi M., Correia D., Pereira N., Salado M., Costa C.M., Lanceros-Mendez S., de la Caba K., Guerrero P. (2023). Sustainable Collagen Blends with Different Ionic Liquids for Resistive Touch Sensing Applications. Acs Sustain. Chem. Eng..

[30] Bayomi M.A., Abanumay K.A., Al-Angary A.A. (2002). Effect of inclusion complexation with cyclodextrins on photostability of nifedipine in solid state. Int. J. Pharm..

[31] Mohan P.R.K., Sreelakshmi G., Muraleedharan C.V., Joseph R. (2012). Water soluble complexes of curcumin with cyclodextrins: Characterization by FT-Raman spectroscopy. Vib. Spectrosc..

[32] Li R., Pavuluri S., Bruggeman K., Long B.M., Parnell A.J., Martel A., Parnell S.R., Pfeffer F.M., Dennison A.J.C., Nicholas K.R. (2016). Coassembled nanostructured bioscaffold reduces the expression of proinflammatory cytokines to induce apoptosis in epithelial cancer cells. Nanomed.-Nanotechnol. Biol. Med..

[33] Li R., Tai M.R., Wu Y.R., Zhou Q.L., Xia Q.Y., Zhong S.Y., Qi Y., Barrow C.J., Williams R.J. (2023). Controlling the supramolecular ordering of fish gelatin via simultaneous assembly with fucoidan resulting in nanostructural modification and enhanced rheological performance. Lwt-Food Sci. Technol..

[34] Warrant R.W., Kim S.-H. (1978). α-Helix–double helix interaction shown in the structure of a protamine-transfer RNA complex and a nucleoprotamine model. Nature.

[35] Nuerxiati R., Mutailipu P., Abuduwaili A., Dou J., Aisa H.A., Yili A. (2021). Effects of different chemical modifications on the structure and biological activities of polysaccharides from *Orchis chusua* D. Don. J. Food Sci..

[36] Tan M.H., Zhao Q.S., Zhao B. (2021). Physicochemical properties, structural characterization and biological activities of polysaccharides from quinoa (*Chenopodium quinoa* Willd.) seeds. Int. J. Biol. Macromol..

[37] Pan Y.C., Sackmann E.K., Wypisniak K., Hornsby M., Datwani S.S., Herr A.E. (2016). Determination of equilibrium dissociation constants for recombinant antibodies by high-throughput affinity electrophoresis. Sci. Rep..

[38] Velazquez-Campoy A., Freire E. (2006). Isothermal titration calorimetry to determine association constants for high-affinity ligands. Nat. Protoc..

[39] Tai M., Cai H., Li R., Jia X., Liu X., Ji H., Zhong S. (2022). Physicochemical properties and immunomodulatory effects of fucoidan from different brown algae. J. Guandong Ocean Univ..

[40] Wei Y., Zhang L., Yu Z.P., Lin K.S., Yang S.F., Dai L., Liu J.F., Mao L.K., Yuan F., Gao Y.X. (2019). Enhanced stability, structural characterization and simulated gastrointestinal digestion of coenzyme Q10 loaded ternary nanoparticles. Food Hydrocoll..

[41] Chen S., Li Q., McClements D.J., Han Y.H., Dai L., Mao L.K., Gao Y.X. (2020). Co-delivery of curcumin and piperine in zein-carrageenan core-shell nanoparticles: Formation, structure, stability and in vitro gastrointestinal digestion. Food Hydrocoll..

[42] Ptak S.H., Sanchez L., Fretté X., Kurouski D. (2021). Complementarity of Raman and Infrared spectroscopy for rapid characterization of fucoidan extracts. Plant Methods.

[43] Li J., Peng J., Huang W.H., Wu Y., Fu J., Cong Y., Xue L.J., Han Y.C. (2005). Ordered honeycomb-structured gold nanoparticle films with changeable pore morphology: From circle to ellipse. Langmuir.

[44] Nikonenko N.A., Buslov D.K., Sushko N.I., Zhbankov R.G. (2005). Spectroscopic manifestation of stretching vibrations of glycosidic linkage in polysaccharides. J. Mol. Struct..

[45] Pasandide B., Khodaiyan F., Mousavi Z.E., Hosseini S.S. (2017). Optimization of aqueous pectin extraction from *Citrus medica* peel. Carbohydr. Polym..

[46] Vollath D., Fischer F.D., Holec D. (2018). Surface energy of nanoparticles—Influence of particle size and structure. Beilstein J. Nanotechnol..

[47] Bartoli E., Fra G.P., Schianca G.P.C. (2011). The oral glucose tolerance test (OGTT) revisited. Eur. J. Intern. Med..

[48] Sakaguchi K., Takeda K., Maeda M., Ogawa W., Sato T., Okada S., Ohnishi Y., Nakajima H., Kashiwagi A. (2016). Glucose area under the curve during oral glucose tolerance test as an index of glucose intolerance. Diabetol. Int..

[49] Banach M., Surma S., Reiner Z., Katsiki N., Penson P.E., Fras Z., Sahebkar A., Paneni F., Rizzo M., Kastelein J. (2022). Personalized management of dyslipidemias in patients with diabetes-it is time for a new approach (2022). Cardiovasc. Diabetol..

[50] Mok Z.H. (2024). The effect of particle size on drug bioavailability in various parts of the body. Pharm. Sci. Adv..

[51] Di J., Gao X., Du Y., Zhang H., Gao J., Zheng A. (2021). Size, shape, charge and "stealthy" surface: Carrier properties affect the drug circulation time *in vivo*. Asian J. Pharm. Sci..

[52] Wu H., Nan J., Yang L., Park H.J., Li J. (2023). Insulin-loaded liposomes packaged in alginate hydrogels promote the oral bioavailability of insulin. J. Control. Release.

[53] Czuba E., Diop M., Mura C., Schaschkow A., Langlois A., Bietiger W., Neidl R., Virciglio A., Auberval N., Julien-David D. (2018). Oral insulin delivery, the challenge to increase insulin bioavailability: Influence of surface charge in nanoparticle system. Int. J. Pharm..

[54] Fan W., Xia D., Zhu Q., Li X., He S., Zhu C., Guo S., Hovgaard L., Yang M., Gan Y. (2018). Functional nanoparticles exploit the bile acid pathway to overcome multiple barriers of the intestinal epithelium for oral insulin delivery. Biomaterials.

